# Targeting Pediatric Obesity Through Gender-Specific Nutritional Strategies: Insights from Dietary Intake and Food Sources

**DOI:** 10.3390/children12121705

**Published:** 2025-12-17

**Authors:** Tadeja Jakus, Breda Prunk Franetič, Tamara Poklar Vatovec

**Affiliations:** 1 Faculty of Health Sciences, University of Primorska, 6310 Izola, Slovenia; 2 Red Cross Health Resort Slovenia, Debeli Rtič, 6280 Ankaran, Slovenia

**Keywords:** childhood obesity, nutrition, dietary, sex-specific

## Abstract

**Highlights:**

**What are the main findings?**

- Significant sex-specific differences were observed in nutrient intake among children. Among overweight or obese children, boys consumed more sugar from isotonic drinks and more fat and sodium from processed meat. Girls exceeded the recommended sugar and total energy intake. Girls also exceeded the Dietary Reference Values (DRVs) for fat by 187%. In addition to processed meat, the method of food preparation contributed to the higher fat intake among girls.

**What are the implication of the main findings?**

- Interventions should be tailored to sex-specific dietary patterns to improve the effectiveness of pediatric obesity prevention and treatment programs.
- Targeted strategies could address portion sizes for both sexes, particularly for protein-rich foods. Boys should be made aware of sugar intake from isotonic drinks and hidden fats in processed meat products. Girls should be encouraged to better understand portion sizes, improve carbohydrate quality, and choose healthier food preparation methods.

**Abstract:**

**Background/Objectives**: Childhood obesity remains a major global health challenge influenced by poor dietary patterns and excessive energy intake. Understanding gender-specific nutritional deviations may improve the effectiveness of preventive and therapeutic interventions. This study aimed to evaluate differences in energy and nutrient intake between boys and girls with overweight or obesity status and to identify the main food sources contributing to these differences. **Methods**: Data from a total of 180 participants (83 boys, 97 girls; 7–18 years) attending the national obesity intervention program Camp My Challenge was analyzed. Anthropometric parameters and dietary intake were assessed using a validated food-frequency questionnaire (OPKP). Intakes were compared with Slovenian dietary reference values (DRVs). Group differences were tested using ANOVA, *t*-tests, and Pearson correlations (*p* < 0.05). **Results**: Boys exhibited higher body mass (79.9 ± 22.6 kg vs. 69.2 ± 19.1 kg; *p* = 0.001) and BMI (30.8 ± 4.8 kg/m^2^ vs. 28.5 ± 4.4 kg/m^2^; *p* = 0.001). Mean energy intake was 2543 ± 1138 kcal/day, exceeding DRV by 16% (t = 3.31, *p* < 0.001). Girls exceeded energy requirements by 24.5% vs. 5.4% in boys (*p* = 0.019). Boys consumed significantly more total fat (106 ± 61 g vs. 85 ± 47 g; *p* = 0.014), saturated fatty acids (34 ± 20 g vs. 27 ± 13 g; *p* = 0.011), protein (119 ± 63 g vs. 98 ± 41 g; *p* = 0.008), and sodium (3628 ± 2086 mg vs. 2852 ± 1520 mg; *p* = 0.005). Girls showed higher sugar intake (208% vs. 166% of DRV; *p* = 0.032), mainly from sweet foods (24%) and fruit (26%), whereas beverages—predominantly isotonic drinks—accounted for 27% of boys’ sugar intake. Sugar intake correlated with waist circumference (r = 0.305, *p* = 0.002) and fat mass (r = 0.272, *p* = 0.007) in girls. Sodium intake exceeded DRV sixfold in both sexes. **Conclusions**: Marked sex-specific dietary disparities exist among children with obesity. Interventions should target high sugar and energy intake in girls and excessive fat, sodium, and sugar-sweetened beverage consumption in boys to enhance the efficacy of pediatric obesity management.

## 1. Introduction

In 2022, more than 390 million children and adolescents aged 5–19 were overweight, including 160 million living with obesity. Additionally, in 2024, approximately 35 million children under age 5 were overweight globally [1]. Obesity in early life is of concern due to health consequences and its influence on later life [2].

Diet plays a central role in the development and prevention of obesity, despite its multifactorial origins involving genetic, psychological and environmental influences. Healthy dietary patterns are characterized by adequate intake of whole grains, dairy products, fish, fruits and vegetables, whereas diets high in processed foods, red meat, sugary beverages, fried foods and fast-food increase obesity risk [3,4]. A systematic review of studies examining the impact of childhood eating habits on obesity risk showed that all studies identified a common eating pattern high in energy density and fat and low in fiber, which is characteristic of a Western diet [5].

Children with obesity often consume sugar-sweetened juices, soft drinks, and sweet, energy-dense foods such as biscuits, pastries, dairy-based desserts, and chips, which are high in fats and/or simple sugars [6]. The Western dietary pattern is also typically poorer in micronutrients, which is a known dietary risk factor that promotes childhood obesity [3]. These trends are also evident among Slovenian children. According to the HBSC study, 24.3% consume sugary beverages daily, with boys doing so more often than girls [7,8]. Fruit and vegetable intake is low: only 39.4% of children eat fruit daily, and just 26.9% eat vegetables [8]. The proportion of children who consume fruit and vegetables at least once a day is higher among younger children and females. Worrying data have directed public health efforts in Slovenia to the development of programs that deal with these problems [9].

Nutrition consumption patterns show notable sex-specific differences throughout the lifespan, including in pediatric populations. Females generally have distinct body composition profiles, energy and nutrient requirements, and metabolic characteristics compared to males. Consequently, the nutrients needed to maintain optimal health vary by age and sex. Within the framework of Dietary Reference Values (DRVs), the tolerable upper intake level (UL) defines the highest amount of a nutrient that can be consumed daily over an extended period without adverse health effects [10]. Studies consistently show sex-based differences in dietary patterns. Boys often exceed the recommended daily intake of nutrients by consuming red meat, sugary drinks, and fast food [11]. Girls tend to follow more varied, healthier eating behaviors, with higher intakes of fruits, vegetables, and plant-based foods, but also tend to eat more frequently (6 or more times per day) [12].

Recognizing these differences in pediatric obesity nutrition is essential for developing targeted nutritional strategies and interventions [11]. This study aimed to identify the nutrients from which children most frequently deviate from national DRVs, determine the main food sources contributing to these deviations, and examine how these patterns differ between boys and girls. By providing detailed, sex-specific insights into dietary inadequacies and excesses, the findings are intended to support the development of more targeted and effective nutritional interventions for pediatric obesity prevention and management.

## 2. Materials and Methods

### 2.1. Study Design

The program, titled “Camp My Challenge,” was implemented at the Red Cross Health Resort of Slovenia at Debeli Rtič as part of the national strategy for tackling childhood obesity, co-funded by the Ministry of Health of the Republic of Slovenia [9]. The study protocol was approved by the Slovenian National Medical Ethics Committee (No. 0120–631/2017/2). Written informed consent was obtained from all subjects who participated in the study. The questionnaire was anonymous, and the data collected was used only for the purposes of the research.

This study was designed as a part of an observational longitudinal intervention evaluation of children participating in a structured 14-day camp program. Over a four-year period, 180 children enrolled in the program. Each child was enrolled in a 14-day structured program that included daily consultations with a pediatrician, psychologist, kinesiologist, physiotherapist or occupational therapist, and a dietitian. Measurements were conducted at five time points—upon arrival (baseline), after the 14-day program, and at 1-month, 6-month and 1-year follow-up—but the present article reports only the baseline data. A detailed description of the study design and implementation has been published elsewhere [13].

### 2.2. Participants

Between 2017 and 2025, a total of 14 camps were organized, involving 180 participants (97 girls and 83 boys), aged between 7 and 18 years. Children were referred to the camp by their personal pediatricians based on predefined criteria aligned with the Slovenian national clinical guidelines for the stepped care approach to childhood obesity treatment at the primary, secondary, and tertiary levels [14]. Eligible participants were children aged 7 to 18 years who met at least one of the following inclusion criteria:

- Obesity: body mass index (BMI) above the 98th percentile for age and sex. The BMI percentile was calculated according to the WHO reference for growth standards for children aged 5–19 years.
- Overweight: BMI between the 91st and 98th percentile, where a prior six-month intervention at the primary care level was unsuccessful.
- Overweight with complications: BMI between the 91st and 98th percentile accompanied by comorbidities such as impaired glucose metabolism (e.g., diabetes, hyperinsulinism), menstrual irregularities, hirsutism in girls, hyperlipidemia (total cholesterol > 6.0 mmol/L), hypertension, hepatic steatosis, sleep-related breathing disorders, or obesity-related orthopedic issues.
- Children with a BMI within the normal range but who experienced a shift of two or more major percentile curves between two preventive check-ups (e.g., from the 25th to the 75th percentile).

Children were excluded if they had chronic diseases (e.g., thyroid disorders), genetic or endocrine conditions affecting growth or metabolism, medications influencing appetite or weight regulation (such as corticosteroids or psychostimulants), physical limitations preventing participation in daily activities, or any acute illness at baseline.

### 2.3. Anthropometric Measurements

Upon arrival at the camp, all participants underwent anthropometric and body composition assessments. Measurements were conducted between 7:00 and 9:00 a.m. under standardized conditions by the same trained dietitian, following an overnight fast.

Height was measured to the nearest 0.1 cm using a Leicester height meter (Invicta Plastics Limited, Oadby, UK), with children standing upright without footwear. Body weight was recorded to the nearest 0.1 kg. BMI was calculated as weight in kilograms divided by height in meters squared (kg/m^2^). The BMI percentiles were calculated according to the WHO reference for growth standards for children aged 5–19 years. Waist circumference was measured in a standing position using a flexible tape measure. Waist-to-height ratio (WHtR) was calculated as waist circumference (cm) divided by height (cm) [15]. Body composition, including total body fat mass and fat-free mass, was assessed using bioelectrical impedance analysis (BIA) with the Tanita BC 418MA device (Tanita Corporation, Arlington Heights, IL, USA), and data were processed using GMON Pro 3.2.1 software provided by the same manufacturer.

### 2.4. Dietary Data Collection

Energy and nutrient intake were assessed using an online food frequency questionnaire (FFQ) [16] available via the Open Platform for Clinical Nutrition (OPKP) at http://opkp.si/ (accessed on 27 November 2025). The questionnaire comprises 16 pages and 61 questions, with content divided into the following nine food groups: milk and dairy products (milk, yogurt, cheese); fats and fatty foods (oils, butter, cream); fruit (raw fruit, cooked fruit, dried fruit, nuts); vegetables (vegetables and legumes); meat, meat products and eggs; cereals and cereal products; potatoes; sweet foods and confectionery; other dishes; and beverages. Children answered questions by selecting from predefined frequency categories regarding the number of meals or frequency of consumption (never; less than once per month; once to three times per month; once a week; two to four times per week; five to six times per week; once per day; two to three times per day; four to five times per day). They also indicated usual portion sizes per meal (measured in cups, tablespoons, pieces, etc.). The questionnaire was completed in collaboration with a dietitian to help ensure accurate reporting of portion size, composition, and frequency of food intake. To assist with estimation, food models and visual aids showing portion sizes of meals and beverages were used [17]. Food, beverage, total energy, and nutrient intake were calculated from the collected food data using OPKP a web-based application (available at https://opkp.si, accessed on 10 October 2025), which is primarily based on the Slovenian food composition table. The average daily energy and macronutrient intake of the children was compared with the recommended daily energy requirements and macronutrient needs [4]. These recommendations were calculated individually for each child, taking into account sex, age, body weight, height, and physical activity level. To assess nutrient adequacy, each individual’s usual intake was compared with the current recommendations for adequate intake and reference intake range defined by NIJZ [4], including the accepted macronutrient distribution range, which was set as a percentage of total energy intake or in g/kg body weight.

### 2.5. Statistical Analysis

The results obtained were processed using different statistical methods. The Kolmogorov–Smirnoff normality test was used to assess the normality of the variables’ distributions in order to determine whether parametric or non-parametric analyses should be applied. One-way Analysis of Variance (ANOVA) was used to examine differences between male and female participants in the measured anthropometric data and dietary intake. The level of statistical significance was set at *p* < 0.05, and results are reported as *F*-values together with corresponding *p*-values. A one-sample *t*-test was used to compare the dietary and energy intake with dietary and energy recommendations. To determine the differences between actual intake and recommendations between the sexes, we used an independent sample *t*-test. Pearson correlations were calculated to evaluate associations among different anthropometric and dietary variables. All data analyses were performed using IBM SPSS 20.0 (IBM Corp., Armonk, NY, USA).

## 3. Results

### 3.1. Subjects’ Characteristics

A total of 180 children aged 7 to 18 years participated in the study, of whom 83 (46.1%) were boys and 97 (53.9%) were girls. Boys had significantly higher values for most anthropometric measures, including body mass, height, waist circumference, fat-free mass, and BMI (all *p* < 0.01) (Table 1).

Gender was negatively correlated with the following body composition parameters: waist circumference (r = −0.352, *p* < 0.001), body mass (r = −0.249, *p* < 0.001), and FFM (r = −0.259, *p* < 0.001), indicating that males had higher values of these parameters.

### 3.2. Energy and Dietary Intake

Average daily energy and nutrient intakes were presented in Table 2. The average energy intake was 2543 ± 1138 kcal/day, and the overall distribution of energy intake from macronutrients was as follows: carbohydrates contributed 49% of total energy intake, fats 33%, and proteins 18%. Boys reported higher intake of all macronutrients (carbohydrates, proteins, and fats) compared to girls. Statistically significant differences were observed for total fat intake (*p* = 0.014), saturated fatty acids (*p* = 0.011), protein intake (*p* = 0.008), and carbohydrate intake (*p* = 0.012). Although differences in total energy intake did not reach statistical significance, boys consistently showed higher absolute intake values.

We also found a link between nutrient intake and body composition (Table 3). Sugar intake was positively linked to waist-to-height ratio (WHtR) and fat mass in both sexes, and in girls, we also found a link to waist circumference. We also showed that high protein intake contributes to lower BMI, fat mass, and waist circumference, but also to lower fat-free mass in both sexes.

### 3.3. Adequacy of Energy and Dietary Intake to the National Recommendations

The analysis revealed that a low proportion of participants met the DRVs for several nutrients (Table 4). Specifically, only 41.7% of participants met the recommended energy intake levels, while 42.2% exceeded and 16.1% were below the recommendations. On average, energy intake exceeded the DRVs by 16% (t = 3.306, *p* < 0.001). Although Table 2 shows that boys had a higher absolute energy intake than girls, when intake was expressed relative to recommendations, girls exceeded the DRVs by 24.5%, compared to only 5.4% in boys (t = –2.359, *p* = 0.019).

Furthermore, a comprehensive analysis of macro- and micronutrient intake relative to the DRVs revealed substantial discrepancies. Some nutrients, including proteins, fats, saturated fatty acids, cholesterol, vitamins C and E, and minerals (Na, Mg, Cl, Fe, Zn, K), were consumed in amounts exceeding the DRVs. In contrast, intakes of certain micronutrients, such as vitamin D, vitamin A and calcium were below the recommended levels in a substantial proportion of participants.

For carbohydrates, only 8.9% of participants met the recommended intake, while 50.6% consumed less than the recommended amount and 40.6% exceeded it (t = 2.795, *p* = 0.006). Girls slightly exceeded the recommended intake, reaching 101.9% of the DRVs, while boys fell short at 95.0% of the DRVs (t = –2.539, *p* = 0.012). Although the average intake of carbohydrates was slightly below the DRVs, the intake of dietary fiber reached recommended levels, with an average of 117% of RV (t = 2.425, *p* = 0.016) among all children. We have shown that girls reach the DRVs (139.7%), while the intake of boys is below the DRVs (91.4%) (t = −5.103, *p* = 0.001). Sugar intake also exceeds the DRVs (t = 8.483, *p* < 0.001), with girls consuming more than boys (t = −2.157, *p* = 0.032).

Protein intake greatly exceeded the DRVs across the total sample, with an average intake reaching 173% of the DRVs (t = 10.040, *p* < 0.001). Both boys and girls had higher protein intake levels than DRVs, with no statistically significant gender difference observed (t = 0.558, *p* = 0.557). However, we showed that the proportion of those exceeding the upper limit of the recommendations was very high (72.3% of boys and 70.1% of girls), suggesting a higher tendency towards excessive protein intake among younger people.

Fat intake substantially exceeded the DRVs in the total sample, with an average intake reaching 250% of the DRVs (t = 4.117, *p* < 0.001). A total of 43.9% of participants consumed fat above the DRVs, with a notably higher proportion among boys (43.4%) compared to girls (29.9%). Saturated fatty acid intake was elevated, averaging 121% of the DRVs (t = 3.602, *p* < 0.001), with more than half of the participants (51.7%) exceeding the upper recommended range. Cholesterol intake was also high, reaching 159% of the DRVs (t = 21.299, *p* < 0.001), and nearly two-thirds of children (67.2%) consumed amounts above the recommended level. Although both sexes exceed the recommended cholesterol intake, the excess is higher for boys than for girls (t = 1.910, *p* = 0.050).

The findings demonstrate that, with the exception of vitamins A and D and calcium, the mean intakes of most assessed micronutrients meet or exceed the established DRVs. However, this apparent adequacy is primarily attributable to a subgroup of individuals exhibiting disproportionately high intake levels (Table 2), thereby skewing the population mean. When examining the proportion of children actually meeting the DRVs, the data reveal a suboptimal pattern: only 29–48% of both girls and boys achieved the recommended intake for critical micronutrients such as vitamin A, vitamin E, and calcium. Sodium intakes were six times higher than recommended levels and slightly higher in boys compared to girls (t = 2.618, *p* = 0.010).

**Figure 1:**
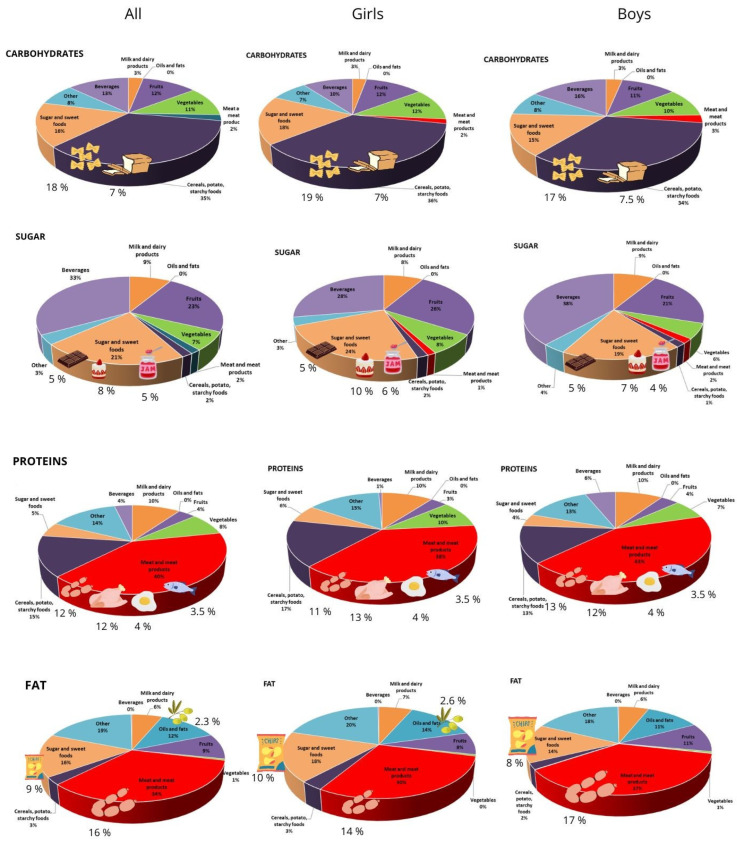
Contribution (%) of the 9 food groups to macronutrient intake in the children.

We subsequently identified the food groups that contributed most substantially to the intake of individual nutrients (Figure 1).

Cereals and cereal-based products were the main source of carbohydrates in the diet (35%). The largest share of cereal-based products and thus carbohydrates was consumed in the form of rice and pasta (18%), followed by bread (14.6%, of which 7% was white bread) and potatoes (4.8%). Together with fruits and vegetables, these food groups accounted for 58% of total carbohydrate intake, with the remainder coming primarily from sweet foods and beverages. The principal sources of sugar were sweet and salty snacks (21%), beverages (33%), and fruit (23%). While beverages represented the primary source of sugar intake, especially in boys (38%), the contribution of sweet and salty snacks (24% vs. 19%) and fruit (26% vs. 21%) was notably higher in girls than in boys. Among beverages, the main contributors to sugar intake were isotonic drinks and vitamin-fortified beverages (21.8%), whereas 100% fruit juices accounted for a smaller proportion (7%). Boys consume considerably more sugar from isotonic drinks compared to girls (27% vs. 16%). In terms of sweet foods, children consume the most sugar from biscuits and cakes (8%), with a higher proportion among girls (10%) than boys (7%). Chocolate (5%) and sweet spreads such as honey and jam (5%) also contribute to sugar intake, with equal proportions between the sexes. Correlation analysis (Table 5) confirmed these patterns, showing a moderate positive relationship between sugar intake and fruit consumption in both boys (r = 0.300, *p* = 0.006) and girls (r = 0.451, *p* < 0.001), as well as a strong association between sugar intake and sweet foods, particularly in girls (r = 0.502, *p* < 0.001).

Meat and meat products were the primary source of protein, accounting for 40% overall (girls: 38%, boys: 43%), but 12% of this protein came from processed meat products (sausages, hot dogs, pâtés), with poultry consumption contributing another significant proportion (12%). Fish consumption accounted for only 3.5% of protein intake for both sexes. Approximately one-quarter of total protein intake originated from plant-based foods, with a slightly higher proportion observed in girls (30%) compared to boys (24%). Correlation data supported this, with a strong positive association between protein intake and meat consumption (boys: r = 0.750, *p* < 0.001; girls: r = 0.682, *p* < 0.001), alongside moderate to strong correlations with cereals in girls (r = 0.614, *p* < 0.001).

It is notable that foods traditionally considered as fat sources, such as oils and butter, contributed only a minor share to total fat intake (12%), of which only 2% of intake is olive oil, which is classified as one of the more nutritionally beneficial sources of fat due to its high content of monounsaturated fatty acids and bioactive compounds. The majority of fats were consumed in hidden form, primarily through meat and meat products (34%), as well as sweet and salty snacks (16%). This is consistent with the strong correlations observed between fat intake and meat products (boys: r = 0.737, *p* < 0.001; girls: r = 0.560, *p* < 0.001). In girls, fat intake was also strongly associated with cereals and potatoes (r = 0.678, *p* < 0.001), indicating that staple carbohydrate-rich foods contributed notably to overall fat intake.

## 4. Discussion

Various approaches have been suggested to prevent and treat adolescent obesity, including dietary interventions with different ratios of carbohydrates, proteins, and fats [18,19]. Intervention programs aimed at reducing obesity in children and adolescents may be more effective if they are gender-specific [20]. There are significant differences between boys and girls in terms of both behavior and risk factors associated with obesity. This study compares nutrient and energy intake according to recommendations and highlights different dietary choices among obese boys and girls, which is important for planning nutritional strategies and designing effective healthy weight loss programs.

The most common cause of obesity in childhood and adolescence is an imbalance in energy, specifically excess caloric intake without adequate caloric expenditure [21]. Our study confirms the findings of other research [22], which, similarly to ours, showed that boys have a higher energy intake than girls. However, in our study, boys exceeded the DRVs by only 5%, while girls exceeded them by 24%. These sex differences are likely related to physiological and behavioral factors. Girls have a lower fat-free mass (FFM) and consequently a lower basal metabolic rate compared with boys of the same age, resulting in lower energy requirements. Evidence shows that FFM is the strongest predictor of resting energy expenditure in children and adolescents, and girls’ lower FFM therefore places them at higher risk of positive energy balance when intake increases even modestly [23]. In addition, several studies have shown that with the onset of adolescence, girls experience a more pronounced decline in physical activity levels than boys, reducing total daily energy expenditure and further contributing to disproportionate energy surplus [20]. Together, the combination of lower FFM, lower basal metabolic rate, and reduced physical activity may help explain why girls in our study exceeded their recommended energy intake to a much greater extent than boys. Further analysis comparing dietary intake revealed that, for energy, carbohydrates, sugar, fat, and fiber, girls deviate more from the recommended values than boys.

Our results highlight important gender-specific differences in carbohydrate and fiber intake. Research indicates that girls often display higher motivation for healthy eating, partly due to sociocultural influences. They are more responsive to health advice and to social or media norms promoting healthy dietary choices [24]. Our findings are consistent with this pattern. Girls met the DRVs for fiber and slightly exceeded the DRVs for carbohydrate intake, whereas boys fell below the recommendations. This aligns with previous research showing that girls generally consume more fruit, vegetables, and wholegrain products than boys [7,8]. In boys, however, we found a positive correlation between vegetable intake and fat intake, suggesting that vegetable dishes are often prepared in ways that increase fat content, most commonly by frying or roasting in butter. This is consistent with evidence from other studies [24] indicating that boys more often choose food based on pleasure, quick energy, or perceived performance benefits, favoring energy-dense foods and preparation methods that enhance palatability.

Excessive sugar intake was evident in both sexes, consistent with European data reporting high intakes of added sugars in children regardless of sex [25]. Despite exceeding DRVs in both sexes, we observed a difference between the sexes, with girls deviating more from DRVs than boys (108% vs. 66%). In addition to contributing to excess energy intake, sugar is also recognized as a risk factor for developing obesity [5]. It is well known that excessive sugar intake contributes to visceral obesity [26]. In our study, we have also shown a statistically significant positive correlation between sugar intake and both waist circumference and waist-to-height ratio. Children have a developed preference for sweet tastes, regardless of gender [27], and this can be associated with high sugar intake. However, hormonal and developmental factors may explain why girls in our sample deviated more strongly from DRVs. Human studies show that fluctuations in reproductive hormones, particularly estrogen and progesterone, affect sweet taste perception and preference [28]. These findings suggest that pubertal hormonal changes may increase susceptibility to higher sugar intake in adolescent girls, which is consistent with the higher relative deviation from DRVs observed in our study. Research shows that the intake of total and added sugars in European countries is high (16–26% of total energy intake) [25], and in our case, we have shown that sugar accounts for 16.4% of daily energy intake. Different countries have different recommendations regarding the intake of total, added, or “free” sugars [25]. There is currently no official recommendation for total sugar intake in children; most recommendations refer to added sugar. The World Health Organization recommends that free sugar intake should not exceed 10% of total energy intake, with a maximum of 5% as the optimal target. Other expert recommendations vary slightly, ranging from 4.4% to 25% of total energy intake [29]. To limit sugar intake, it is essential to identify its sources in food. Studies show that the main source of sugar in the diet is sweet products, followed by fruit, beverages, and dairy products [25]. In our case, the order is slightly different: the main source of sugar is beverages, followed by fruit and sweet products, with proportions varying slightly between the sexes. It is important to note that products in these different food categories have different nutritional densities and therefore do not play the same role in the diet. For example, soft drinks, which are the main problem in terms of sugar intake among Slovenian children, especially boys, primarily contain sugar and small amounts of nutrients beneficial to health. Our study showed that as much as 27% of boys’ total sugar intake comes from isotonic drinks (i.e., sports drinks). This is consistent with data from other studies, which estimate that sports drinks comprise about 26% of total sugar-sweetened beverage intake in adolescents [30]. Sports and isotonic drinks are often marketed as healthy choices because they contain vitamins and minerals and provide electrolyte replenishment; they also promote. However, many sports drinks contain high amounts of sugar, which increases the risk of obesity, type 2 diabetes, and dental caries. Fruit, by contrast, provides not only sugar but also fiber and vitamins, and girls consume more fruit, so their intake of these nutrients is higher. Although fruit is considered a healthy food choice, children need to be educated about fruit selection and portion sizes due to their preference for sweet foods. Research shows that children prefer to eat sweet fruits (such as bananas and watermelons), dried fruits, smoothies, and similar foods [27]. However, these types of fruit are rich in sugars, so consuming large quantities in your diet can increase your intake of free sugars and have a positive effect on your energy balance, which may lead to obesity in the long term [31].

These findings suggest that dietary strategies should emphasize improving the quality of carbohydrate sources in girls, particularly regarding sweet foods, fruit choices, and portion sizes. For boys, the focus should be on increasing fiber intake through whole grains, fruits, and vegetables. Strategies to reduce free sugar intake need to be developed for both sexes, but it is especially important to raise awareness among boys about the hidden sugar content in beverages. Our study showed that up to 27% of boys’ total sugar intake comes from isotonic drinks (i.e., sports drinks). This highlights the importance of targeted interventions in this population group.

Protein intake was well above the recommended daily intake for both sexes (169–177%). Research shows a strong tendency to consume protein during weight loss, mainly due to the belief that it helps increase muscle mass and reduce fat mass [32]. Evidence suggests that these trends develop early in childhood, likely due in large part to protein advertising [33]. In the future, greater attention should be given to planning both the quantity and source of protein in the diet. Our findings indicate that the main sources of protein in children’s diets are meat (particularly poultry) and meat products. Processed meat products are considered lower-quality protein sources because, in addition to essential amino acids, they typically contain high levels of saturated fats, sodium, and additives (such as nitrates and nitrites), which have been linked to increased risks of cardiovascular disease, certain cancers, and higher overall mortality [34]. On the other hand, the proportion of protein derived from fish is low (3.5%). Fish is a rich source of omega-3 fatty acids, which are important during adolescence. Research also shows that the trend of meat consumption is higher among men, while women consume legumes more frequently in addition to meat [35]. In our study, we also demonstrated that protein intake in boys was derived mainly from meat and processed meat products, whereas in girls, plant sources accounted for approximately 30% of total protein intake. We further confirmed that plant-based foods contribute to protein intake in girls by correlating protein intake with vegetables (including legumes) and cereals. In both sexes, we observed a negative correlation between protein intake and fat-free mass (FFM). This result can be explained by the fact that excess protein also contributes to a positive energy balance and, in the absence of exercise—which is often the case with obesity, especially in girls [36]—does not contribute to an increase in muscle mass. Finally, when energy demand is low, excess protein can be converted to glucose (via gluconeogenesis) or ketone bodies and contribute to a positive energy balance, which is undesirable if weight loss is the goal [32]. Studies show that girls are less responsive to physical activity interventions and more responsive to dietary changes (not least because they are influenced by protein advertising) [21,26], it is important to make them aware of the appropriate protein intake in their diet.

High consumption of meat and processed meat products significantly increases the intake of total fats, cholesterol, and saturated fats. In our study, we found that girls exceeded the dietary reference values (DRVs) for fat intake by up to 186%, while boys exceeded them by 106%. The consumption of energy-dense, high-fat foods is recognized as a dietary pattern contributing to obesity [5], although in our study we did not find any association between fat intake and body composition. Given the magnitude of these deviations, it is important to note that FFQ-based assessments may overestimate or underestimate the intake of energy-dense foods, particularly those containing hidden fats, and some of the extreme values—especially among girls—may partly reflect misclassification rather than true habitual intake. FFQs rely on retrospective recall, fixed portion-size categories, and subjective estimation, which may obscure day-to-day variation in fat consumption and reduce the sensitivity needed to detect true associations with body composition. Therefore, the absence of a significant association in our findings should be interpreted with caution, as it may be partly attributable to limitations of the dietary assessment method rather than the absence of a biological relationship. High fat intake also leads to higher daily energy intake due to the high energy value of fats, which in our case exceeded recommended levels. We found that fat intake was high mainly because of hidden fats in processed meat products and snacks, as the proportion of intake from pure fats (oil, butter, margarine) was relatively low (20% of total fat). Fat intake from olive oil and olives was only 2%, although this source is considered nutritionally beneficial due to its high content of monounsaturated fatty acids and bioactive compounds [37]. In addition, fat intake is strongly influenced by the method of food preparation. In one of our studies, we found that more than half of children eat fried foods several times a week [13]. We also found a correlation between fat intake and starchy foods in both sexes, resulting from the method of food preparation (e.g., fried potatoes, fried dough, fried bread slices). The starch–fat link was stronger in girls than in boys, while the meat product–fat link was stronger in boys. Therefore, in addition to raising general awareness about hidden fats, it would be sensible for future nutritional strategies to focus on reducing meat product consumption in boys and increasing awareness about food preparation methods (fried foods) in girls.

Micronutrients, including vitamins and minerals, play a crucial role in children’s growth and development, affecting bone health, immune function, and cognitive performance. Deficiencies during this sensitive period can have long-term health consequences and increase the risk of obesity-related complications [3]. Ensuring adequate intake of these nutrients is therefore essential, and interventions targeting pediatric populations should address both macronutrient balance and the optimization of micronutrient status. Analysis of micronutrient intake showed that, although average values met or exceeded DRVs for most vitamins and minerals, a significant proportion of children did not achieve adequate intake of key nutrients such as vitamin A, vitamin D, and calcium. These deficiencies are consistent with previous research linking suboptimal micronutrient intake to the risk of pediatric obesity [3]. Furthermore, sodium intake was alarmingly high, particularly in boys, reaching more than six times the recommended levels, which mirrors the findings of other European pediatric cohorts [11]. However, although our findings are consistent with results from other international studies, caution is warranted when interpreting the exceptionally high sodium values, as FFQ-based assessments may overestimate intake due to difficulties in capturing hidden salt in processed foods and added salt in meals [38]. In contrast, girls were more likely to exceed recommendations for vitamin C, reflecting their higher fruit intake [8]. These results reinforce the importance of considering both excesses and inadequacies in micronutrient intake when designing interventions, and they underscore the need for gender-tailored strategies—for instance, reducing sodium and hidden sources of salt in boys’ diets, while focusing on improving vitamin D and calcium intake across both sexes.

The findings in our study should be interpreted with caution due to several limitations inherent to the study design and data collection methods. Although the FFQ used in this study has been validated for the Slovenian population, FFQ-based dietary reporting is subject to several well-known limitations. These include reliance on memory, difficulties in estimating portion sizes, and the tendency toward social desirability bias, which may lead to both under- and over-reporting of specific foods. Such limitations are particularly relevant at the extremes of intake, where very high or very low estimates may reflect misclassification rather than true habitual consumption. In the context of our findings, the large deviations from DRVs—especially the substantial excesses of total fat, saturated fat, sodium, and protein—should therefore be interpreted cautiously. In addition, the structure of the questionnaire (e.g., limited set of categories, predetermined portions) may also contribute to higher values. A further limitation concerns the lack of systematically collected data on pubertal development (e.g., Tanner staging). Although each child underwent clinical evaluation by a pediatrician, and any noticeable deviations in growth or pubertal development were taken into account in their individual treatment plan, pubertal status was not recorded in a standardized manner for research purposes. As a result, we were unable to adjust analyses for pubertal maturation, which is known to influence body composition, metabolic profile, and nutritional requirements.

## 5. Conclusions

Understanding the diverse gender-specific nutritional requirements and dietary habits is essential for guiding dietitians and clinicians in tailoring nutritional interventions for pediatric patients.

Our findings indicate that weight management programs for children and adolescents should adopt a gender-specific approach. Boys in our sample had higher body mass, waist circumference, fat-free mass, and energy intake, with diets characterized by higher absolute intakes of fats (including saturated fatty acids), proteins, cholesterol, and sodium. Interventions for boys should therefore focus on reducing sugar from isotonic drinks and total and saturated fat intake (from meat and meat products) and increasing dietary fiber (whole grain products, vegetables, fruits) and micronutrients that are often below recommended levels (e.g., vitamin D, calcium, magnesium).

Girls, however, exceeded recommended energy intake to a greater extent relative to their needs and had a higher proportional intake of sugars, particularly from sweet foods and fruit. For girls, intervention strategies should focus on moderating total sugar intake, improving the quality of carbohydrate sources, and maintaining adequate but not excessive fruit consumption. Additionally, both groups would benefit from increased intakes of micronutrients that were insufficient in a large proportion of participants (vitamin D, vitamin A, calcium), but the approaches to achieving this should reflect the dominant food sources in each gender’s diet.

Overall, gender-tailored nutritional counseling—combined with physical activity programs adapted to differences in body composition—may enhance the effectiveness of obesity management in youth.

## Figures and Tables

**Table 1:** Children anthropometric characteristics by gender.

Children Characteristics	Total *n* = 180	Boys *n* = 83	Girls *n* = 97	*p*	F
Age (years)	11.5 ± 2.4	11.8 ± 2.2	11.3 ± 2.6	0.119	2.452
Body mass (kg)	74.2 ± 21.4	79.9 ± 22.6	69.2 ± 19.1	**0.001**	11.801
Body height (cm)	156.8 ± 13.3	159.7 ± 13.5	154.2 ± 12.7	**0.005**	7.924
WC (cm)	94.5 ± 13.1	99.5 ± 12.3	90.2 ± 12.3	**0.001**	25.106
Fat mass (kg)	28.1 ± 10.8	30.4 ± 11.1	26.2 ± 10.2	**0.010**	6.859
Fat percentage (%)	37.3 ± 6.3	37.6 ± 7.2	36.9 ± 5.5	0.474	0.514
Fat free mass (kg)	46.0 ± 12.6	49.5 ± 14.3	43.0 ± 10.1	**0.001**	12.833
BMI (kg/m^2^)	29.6 ± 4.7	30.8± 4.8	28.5 ± 4.4	**0.001**	11.536
WHtR (cm/cm)	0.61 ± 0.06	0.62 ± 0.05	0.60 ± 0.06	**0.017**	5.792

Results are presented as mean ± standard deviation. WC, waist circumference; BMI, body mass index; *n*, the number of subjects; %, percentage of subjects; WHtR, waist to height ratio. Bold values indicate statistically significant results (*p* < 0.05).

**Table 2:** Children dietary and energy intakes by gender.

Variable		All (*n* = 180)	Boys (*n =* 83)	Girls (*n =* 97)	*p*	F
Daily Energy and Macronutrient Intake						
Energy intake	kcal/day	2543 ± 1138	2711 ± 1148	2399 ± 1115	0.066	3.421
CHO	g/day	309 ± 146	313 ± 137	306 ± 154	0.751	0.101
	% Energy intake	49 ± 9	47 ± 11	51 ± 8	**0.012**	0.751
Sugar	g/day	103.7 ± 74.8	105.5 ± 75.1	102.1 ± 74.9	0.088	0.768
	% Energy intake	16.4 ± 7.7	15.7 ± 7.7	16.9 ± 7	0.959	0.329
Proteins	g/kg BM	1.6 ± 0.8	1.6 ± 1.0	1.5 ± 0.7	0.573	0.318
	g/day	107 ± 53	119 ± 63	98 ± 41	**0.008**	7.134
	% Energy intake	18 ± 4	18 ± 4	17 ± 4	0.313	1.024
Fats	g/day	95 ± 55	106 ± 61	85 ± 47	**0.014**	6.111
	% Energy intake	33 ± 8	34 ± 9	31 ± 7	**0.034**	4.590
SFAs	g/day	30 ± 17	34 ± 20	27 ± 13	**0.011**	6.523
	% Energy intake	11 ± 3	11 ± 3	10 ± 3	0.176	1.847
Cholesterol	mg/day	478 ± 301	525 ± 357	438 ± 238	**0.050**	3.877
Fiber	g/day	28 ± 15	28 ± 15	29 ± 15	0.595	0.283
Vitamin A	µg/day	861 ± 674	911 ± 803	819 ± 540	0.365	0.825
Vitamin D	µg/day	3 ± 2	4 ± 3	3 ± 2	0.255	1.305
Vitamin E	µg/day	12 ± 7	13 ± 7	12 ± 6	0.283	1.161
Vitamin C	mg/day	167 ± 95	172 ± 92	296 ± 212	0.597	0.280
Cl	mg/day	5084 ± 2799	5714 ± 3149	4545 ± 2346	**0.005**	8.103
K	mg/day	3296 ± 1430	3443 ± 1486	3171 ± 1376	0.205	1.615
Ca	mg/day	1102 ± 558	1152 ± 578	1059 ± 539	0.269	1.230
Mg	mg/day	335 ± 145	348 ± 150	324 ± 141	0.279	1.178
Fe	mg/day	20 ± 9	21 ± 8	20 ± 9	0.268	1.233
Zn	mg/day	12 ± 5	12 ± 6	11 ± 5	0.097	2.778
Na	mg/day	3210 ± 1840	3628 ± 2086	2852 ± 1520	**0.005**	8.279

CHO, carbohydrates; SFAs, saturated fatty acids. Results are presented as mean ± standard deviation. Bold values indicate statistically significant results (*p* < 0.05).

**Table 3:** Correlation between nutrient intake and body composition.

			FFM (kg)	WC (cm)	BMI (kg/m^2^)	FM (%)	WHtR
Sugar (g/day)	M	r	0.059	0.141	0.108	0.277	0.200
		*p*	0.596	0.202	0.331	**0.011**	**0.070**
	F	r	0.103	0.305	0.226	0.272	0.239
		*p*	0.315	**0.002**	**0.026**	**0.007**	**0.019**
Proteins (g/kg BW)	M	r	−0.334	−0.324	−0.340	−0.092	−0.197
		*p*	**0.002**	**0.003**	**0.002**	0.406	**0.074**
	F	r	−0.498	−0.339	−0.370	−0.227	−0.020
		*p*	**<0.001**	**<0.001**	**<0.001**	**0.025**	0.849
CHO (g/day)	M	r	0.061	0.141	0.074	0.070	0.074
		*p*	0.584	0.204	0.503	0.530	0.508
	F	r	0.062	0.237	0.214	0.232	0.245
		*p*	0.548	**0.019**	**0.036**	**0.022**	**0.016**
Fat (g/day)	M	r	0.054	0.072	0.092	0.048	0.019
		*p*	0.627	0.515	0.409	0.669	0.864
	F	r	0.044	0.169	0.134	0.143	0.092
		*p*	0.667	0.097	0.190	0.163	0.372

M, male; F, female; CHO, carbohydrates; WC, waist circumference; BMI, body mass index; WHtR, waist to height ratio; FFM, fat free mass; FM, fat mass. Bold values indicate statistically significant results (*p* < 0.05).

**Table 4:** Percentages of children meeting and not meeting recommendations for main fatty acids by gender.

	All (*n* = 180)					Boys (*n* = 83)				Girls (*n* = 97)				
Variable	% RV *	t (*p*) ^1^	% MR	% BR	% AR	% RV *	% MR	% BR	% AR	% RV *	% MR	% BR	% AR	t (*p*) ^2^
**Daily energy and macronutrient intake**														
Energy intake	116	3.306 **(<0.001)**	41.7	16.1	42.2	105.4	14.5	43.4	42.2	124.5	17.5	40.2	42.3	−2.359 **(0.019)**
CHO	99	2.795 **(0.006)**	8.9	50.6	40.6	95.0	8.4	57.8	33.7	101.9	9.3	44.3	46.4	−2.539 **(0.012)**
Sugar	188	8.483 **(<0.001)**	25.6	-	74.4	165.6	24.1	-	75.9	208.2	26.8	-	73.2	−2.157 **(0.032)**
Proteins	173	10.040 **(0.001)**	11.1	17.8	71.1	176.8	7.2	20.5	72.3	168.8	14.4	15.5	70.1	0.558 (0.557)
Fats	250	4.117 **(<0.001)**	11.7	44.4	43.9	206.7	24.1	32.5	43.4	286.7	24.7	45.4	29.9	−0.435 (0.664)
SFAs	121	3.602 **(<0.001)**	48.3	48.3	51.7	125.2	49.4	-	50.6	117.8	47.4	-	52.6	0.689 (0.491)
Cholesterol	159	21.299 **(<0.001)**	32.8	32.8	67.2	175.2	32.5	-	67.5	145.9	33	-	67	1.910 **(0.050)**
Fiber	117	2.425 **(0.016)**	50.6	49.4	50.6	91.4	62.7	37.3	-	139.7	61.9	38.1	-	−5.103 **(0.001)**
**Daily micronutrient intake**														
Vitamin A	91	−1.974 **(0.025)**	28.9	71.1	0	89.8	28.9	71.1	-	91.8	28.9	71.1	-	−0.177 (0.860)
Vitamin D	16	−96.511 **(<0.001)**	0	100	0	17.5	0	100	0	15.6	0	100	0	1.126 (0.262)
Vitamin E	102	0.022 (0.491)	42.8	57.2	0	97.0	36.1	63.9	-	106.0	48.5	51.5	-	−1.039 (0.300)
Vitamin C	217	−11.730 **(<0.001)**	83.3	16.7	0	206.3	80.7	19.3	-	227.4	85.6	14.4	-	−0.979 (0.329)
Cl	636	20.507 **(<0.001)**	100	0	0	707.5	100	-	-	574.1	100	-	-	2.508 **(0.011)**
K	178	−13.434 **(<0.001)**	89.7	13.3	0	183.0	90.4	9.6	-	173.3	83.5	16.5	-	0.833 (0.406)
Ca	99	−0.508 (0.306)	38.3	61.7	0	101.8	43.4	56.6	-	96.7	34.0	66	-	0.661 (0.510)
Mg	117	−3.134 **(0.001)**	57.8	42.2	0	113.3	55.4	44.6	-	120.0	59.8	40.2	-	−0.800 (0.425)
Fe	165	−11.512 **(<0.001)**	82.8	17.2	0	183.4	88.0	12.0	-	149.6	78.4	21.6	-	−2.569 **(0.011)**
Zn	147	9.185 **(<0.001)**	72.2	27.8	0	133.0	59.0	41.0	-	158.6	83.5	16.5	-	−2.569 **(0.011)**
Na (mg)	605	19.518 **(<0.001)**	0	0	100	677.7	0	0	100	543.0	0	0	100	2.618 **(0.010)**

The percentage for inadequacy was calculated by comparing with NIJZ recommendations. RV- * Dietary reference values [4]. MR, percentage meeting recommendations; BR, percentage below recommendations; AR, percentage above recommendations (AR); CHO, carbohydrates; SFAs, saturated fatty acids; ^1^—statistically significant difference between recommended intake and actual intake; ^2^—statistically significant difference between gender. Bold values indicate statistically significant results (*p* < 0.05). If BR is not reported: intake ≤ recommended values. If AR is not reported: intake ≥ recommended values.

**Table 5:** Correlation between nutrient intake and food groups.

			Cereals, Potato	Fruits	Oils and Fats	Milk and Dairy Products	Meat, Eggs and Meat Products	Sweet and Salty Snacks	Vegetables
Sugar (g/day)	M	r	0.245	0.300	0.179	0.277	−0.096	0.365	0.106
		*p*	**0.026**	**0.006**	0.105	**0.011**	0.390	**<0.001**	0.342
	F	r	0.335	0.451	0.266	0.134	0.171	0.502	−0.003
		*p*	**<0.001**	**<0.001**	**0.009**	0.192	0.093	**<0.001**	0.974
CHO (g/day)	M	r	0.781	0.240	0.340	0.420	0.083	0.551	0.329
		*p*	**<0.001**	**0.029**	**0.002**	**<0.001**	0.453	**<0.001**	**0.002**
	F	r	0.856	0.365	0.492	0.240	0.377	0.537	0.176
		*p*	**<0.001**	**<0.001**	**<0.001**	**0.018**	**<0.001**	**<0.001**	0.085
Proteins (g/day)	M	r	0.232	0.045	0.619	0.184	0.750	0.243	0.167
		*p*	**0.035**	0.630	**<0.001**	0.096	**<0.001**	**0.027**	0.132
	F	r	0.614	0.113	0.551	0.394	0.682	0.333	0.243
		*p*	**<0.001**	0.271	**<0.001**	**<0.001**	**<0.001**	**<0.001**	**0.016**
Fat (g/day)	M	r	0.377	0.059	0.912	0.422	0.737	0.487	0.297
		*p*	**<0.001**	0.595	**<0.001**	**<0.001**	**<0.001**	**<0.001**	**0.006**
	F	r	0.678	0.333	0.857	0.392	0.560	0.433	0.029
		*p*	**<0.001**	**<0.001**	**<0.001**	**<0.001**	**<0.001**	**<0.001**	0.775

M, male; F, female; CHO, carbohydrates; Bold values indicate statistically significant results (*p* < 0.05).

## Data Availability

The results will be communicated to the participants and other relevant groups via publications (open access will be prioritized) and presentations, including webinars.
